# A Natural Variation of Fumonisin Gene Cluster Associated with Fumonisin Production Difference in *Fusarium fujikuroi*

**DOI:** 10.3390/toxins11040200

**Published:** 2019-04-03

**Authors:** Sharmin Sultana, Miha Kitajima, Hironori Kobayashi, Hiroyuki Nakagawa, Masafumi Shimizu, Koji Kageyama, Haruhisa Suga

**Affiliations:** 1 The United Graduate School of Agricultural Science, Gifu University, Gifu 501-1193, Japan; sharmin19822011@gmail.com; 2 Faculty of Applied Biological Sciences, Gifu University, Gifu 501-1193, Japan; l8022024@edu.gifu-u.ac.jp (M.K.); o8022036@edu.gifu-u.ac.jp (H.K.); shimizma@gifu-u.ac.jp (M.S.); 3 Faculty of National Food Research Institute, NARO, Tsukuba 305-8642, Japan; hironkgw@affrc.go.jp; 4 River Basin Research Center, Gifu University, Gifu 501-1193, Japan; kageyama@green.gifu-u.ac.jp; 5 Life Science Research Center, Gifu University, Gifu 501-1193, Japan

**Keywords:** rice bakanae, polyketide mycotoxin, gene diversity, transcriptional regulator, pre-termination

## Abstract

*Fusarium fujikuroi*, a member of the *Fusarium fujikuroi* species complex, stands out as a rice bakanae disease pathogen with a high production of gibberellic acid. Not all, but some *F. fujikuroi* strains are known to produce a carcinogenic mycotoxin fumonisin. Fumonisin biosynthesis is dependent on the FUM cluster composed of 16 *FUM* genes. The FUM cluster was detected not only from a fumonisin producing strain, but also from a fumonisin nonproducing strain that does not produce a detectable level of fumonisin. Genetic mapping indicated the causative mutation(s) of fumonisin nonproduction is present in the FUM cluster of the fumonisin nonproducing strain. Comparative analyses of *FUM* genes between the fumonisin producing and the nonproducing strains and gene complementation indicated that causative mutation of fumonisin nonproduction is not a single occurrence and the mutations are distributed in *FUM21* and *FUM7*. Our research revealed a natural variation in the FUM cluster involving fumonisin production difference in *F. fujikuroi*.

## 1. Introduction

*Fusarium fujikuroi*, a member of the *Fusarium fujikuroi* species complex (*Ff* complex), causes rice bakanae disease by high production of gibberellin in a plant. Beside gibberellin, *F. fujikuroi* is known to produce other secondary metabolites such as fumonisin, moniliformin, fusarins, fusaric acid, and beauvericin [1,2]. Fumonisin is a carcinogenic mycotoxin, and its production has been detected from 15 species of *Ff* complex [1,3,4,5] and *Aspergillus niger* [6]. 

Fumonisin causes various kinds of diseases such as leukoencephalomalacia in horses [7], pulmonary edema in swine [8], and possibly human esophageal cancer [9] and neural tube defects [10]. Fumonisins are also phytotoxic by inhibiting acyl CoA-dependent ceramide synthase in plant cells [11]. The most abundant naturally occurring fumonsin is FB_1_ [12].

*Fusarium proliferatum* and *F*. *fujikuroi* are considered potential fumonisin producers in rice [13] and are the most closely related species. They infrequently produce interspecific hybrids, but most of the natural isolates can be differentiated by molecular markers and chemotaxonomic criteria [14,15]. A majority of strains in *F. proliferatum* produce fumonisin, while fumonisin production has been detected only in partial strains of *F. fujikuroi*. Most especially, frequency of fumonisin production of *F. fujikuroi* from rice is comparatively low: 13 out of 31 strains (41.9%) from rice in the Philippines [13], 27 out of 38 strains (71.0%) from rice in Korea [16], and 33 out of 66 strains (50.0%) from rice in Japan [17]. Only a small amount of fumonisin was detected in a *F. fujikuroi* strain IMI58289 from rice in Taiwan, and gibberellin production of that strain has been intensively studied [18]. Recently, it was revealed that fumonisin producing strains and nonproducing strains in *F. fujikuroi* are phylogenetically different [19,20]. Suga et al. [19] designated fumonisin producer as F-group and nonproducer as G-group. It was demonstrated that G-group has higher gibberellin producibility than F-group and showed typical bakanae symptoms [19].

*F. fujikuroi* was the most frequent isolate in rice from 10 Asian countries [21]. Fumonisins have been found as common contaminants in maize-based foods and feed in the United States of America, China, Europe, South America, and Africa [22,23,24,25]. The frequency and production level of fumonisin in *F. fujikuroi* are varied in the previous publication [13,16,26]. Bolton et al. [26] detected 0.9–2403 ppm FB_1_ in 50 grape isolates with rice culturing for seven days at 27 °C. Cruz et al. [13] detected 0.4–224 ppm FB_1_ in 18 out of 31 rice isolates by culturing with fumonisin-inducing liquid medium for 7 days at 20 °C. Lee et al. [16] detected 0.5–12.9 ppm FB_1_ in 11 corn isolates by rice culturing. Various production level of fumonisin is partly attributed to a different assay method and different growth media used in the individual investigations. Therefore, direct comparisons among previous publications are difficult. However, the possibility of fumonisin production difference in *F. fujikuroi* from different crops have been implicated [19,20]. 

Fumonisin biosynthetic gene (FUM) cluster consisting of 16 *FUM* genes spanning 50 kbp in the genome were revealed on *Fusarium verticillioides* as a member of *Ff* complex [27]. The *FUM* genes were detected from both fumonisin producing and nonproducing *F. fujikuroi* strains [20]. The fumonisin biosynthesis starts with a polyketide synthesis by *FUM1*. Subsequently, it was modified by other genes: *FUM6*, *FUM7*, *FUM8*, *FUM3*, *FUM10*, *FUM11*, *FUM2*, *FUM13*, *FUM14*, and *FUM15* (Table 1). Additional *FUM16, FUM17, FUM18*, and *FUM19* were recognized as *FUM* genes based on their colocation and coregulation with other *FUM* genes [27,28], though disruption of each gene did not alter fumonisin production [28,29]. *FUM21* encodes a Zn(II)-2Cys6 DNA binding transcription factor that positively regulates other *FUM* gene expressions [30]. *FUM21* disruption lacked *FUM1* and *FUM8* expression at least in *F. verticillioides* and failed to produce FB_1_ [30]. Information of the gene polymorphisms affecting fumonisin producibility is important for accurately assessing the fumonisin production risk of *F. fujikuroi*. It was revealed that a low level of expression of *FUM21* is the cause of low fumonisin production in the *F. fujikuroi* strain IMI58289 [18]. However, the causative mutation of fumonisin nonproduction may be varied in *F. fujikuroi*, as a large deletion in the FUM cluster was reported in *Fusarium musae* (previous *F. verticillioides*) [31]. A partial deletion of FUM cluster was also reported in *F. fujikuroi* strain FGSC8932, though its fumonisin production has not been investigated [32]. In this study, we revealed a new type of causative mutation of fumonisin nonproduction in *F. fujikuroi*. The cause is not a single mutation and the causes are distributed in *FUM21* and *FUM7* in the FUM cluster.

## 2. Results

### 2.1. Linkage Analysis of Fumonisin Nonproduction

In total, 100 progenies were obtained from crossing between fumonisin producer Gfc0825009 (G9) and nonproducer Gfc0801001 (G1) (Table S1). Five SNP markers including FUM1_G423A and FUM18_G51T in the FUM cluster [19], and MAT types were used for linkage analysis. Fifty-eight progenies showed identical haplotypes with either of the parental strains and were considered as possibly conidially derived colonies. The remaining 42 progenies showed a different haplotype from the parental strains and were considered true progenies. Twenty-four and 18 progenies were fumonisin producers and nonproducers, respectively. All fumonisin producers showed G9 type and all fumonisin nonproducer showed G1 types in FUM1-G423A and FUM18-G51T markers. These results suggested that the causative mutation of fumonisin nonproducibility in G1 strain is present in the FUM cluster.

### 2.2. Sequence Comparison of the FUM Cluster

The FUM cluster of G1 and G9 strains was sequenced and the *FUM* genes were compared (Figure S1, Table 1). The distribution and the direction of *FUM* genes in G1 strains were the same as in G9 strain. High homology was observed in the nucleotide (98.2–99.6%) and the amino acid (97.5–99.8%) sequences though *FUM17* could not be compared because pre-termination was observed in both strains (Table 1). It was impossible to identify the causative mutations of fumonisin nonproduction from sequence comparison although some amino acid substitutions, insertions, and deletions were detected in G1 strain (Table 1).

### 2.3. FUM Gene Expression

**Figure 1:**
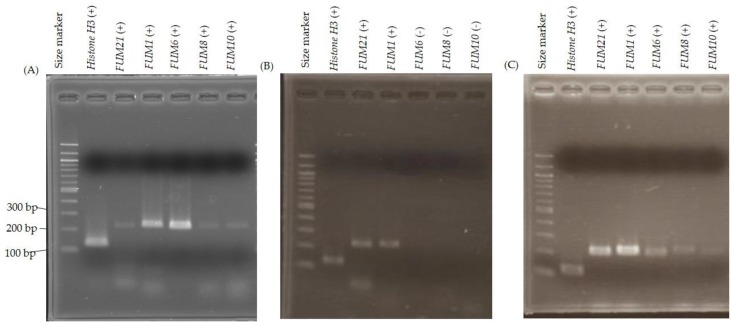
RT-PCR result of (**A**) Gfc0825009, (**B**) Gfc0801001, and (**C**) FfT21FUMKOD (#2). RT-PCR product was subjected to 2% agarose gel electrophoresis. (+) means that the expected size of RT-PCR product was detected, (−) means not detected. *HistoneH3* was used as a positive control.

Expression of *FUM21*, *FUM1*, *FUM6*, *FUM8*, and *FUM10* in G1 and G9 strains were investigated by RT-PCR. The expected size of DNA (Table S2) was detected in all genes of G9 strain while it was detected only in *FUM21* and *FUM1* of G1 strain (Figure 1). RT-PCR was conducted in triplicate culturing and the results were same. These results suggested that some of the essential *FUM* genes are not transcribed in G1 strain under fumonisin production conditions.

### 2.4. Fumonisin Production Recovery by Gene Complementation

**Figure 2:**
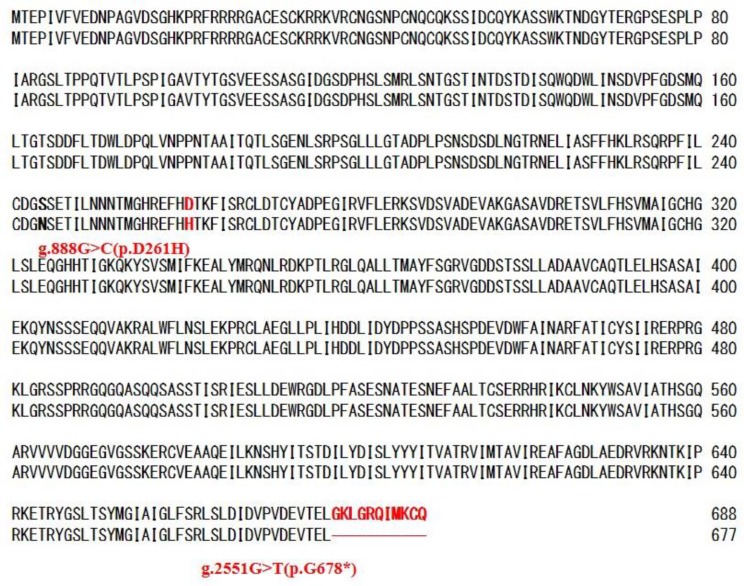
Comparison of the amino acid sequences of FUM21 of fumonisin producing strain Gfc0825009 (**upper**) and fumonisin nonproducing strain Gfc0801001 (**lower**).

**Figure 3:**
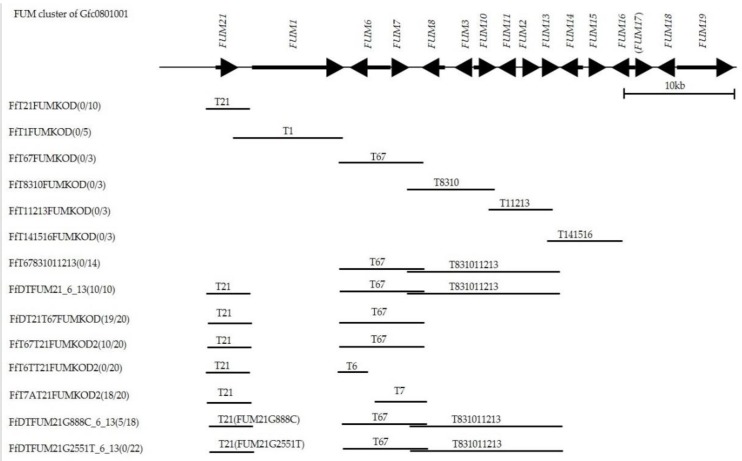
The results of *FUM* gene complementation in Gfc0801001 by transformation. The number of fumonisin producing transformants*/*the number of investigated transformants are indicated in parenthesis after the transformant names. Fumonisin production was determined by the detection of fumonisin by ELISA (detection limit is 0.22 ppm). “T” is the DNA fragment from fumonisin producing strain Gfc0825009. *FUM17* in both Gfc0801001 and Gfc0825009 are pseudogene and, therefore, indicated in parenthesis. *FUM16*, *FUM17*, *FUM18* and *FUM19* are known to be not essential for fumonisin biosynthesis [28,29].

Failure of detection of RT-PCR product from plural *FUM* genes suggested that *FUM21* encoding a transcription factor may be defective in G1 strain, although its RT-PCR product was detected (Figure 1). One amino acid substitution (p.D261H) and a pre-termination (p.G678*) that lacks 11 amino acids at C-terminal of FUM21 were present in G1 strain (Figure 2 and Table 1). We assumed that these mutations dysfunction FUM21 in G1 strain, and it was conducted that *FUM21* of G9 strain was transformed into G1 strain (FfT21FUMKOD in Figure 3). Ten transformants were created, and full-length integration of the T21 fragment that contained *FUM21* and its flanking region was confirmed by PCR (Figure S2). In addition, RT-PCR showed the positive conversion of *FUM6*, *FUM8*, and *FUM10* expression in a transformant, FfT21FUMKOD (#2) (Figure 1). However, fumonisin production recovery was not observed in all transformants (FfT21FUMKOD in Figure 3). Complement transformants were further created in G1 strain with T1 (containing *FUM1*), T67 (containing *FUM6* and *FUM7*), T8310 (containing *FUM8* to *FUM10* region), and T141516 (containing *FUM14* to *FUM16* region) fragment from G9 strain, but fumonisin production recovery was observed in none of them (Figure 3). Fumonisin production recovery was also not observed in simultaneous transformants with T67 and T831011213 (Figure 3). However, fumonisin production was detected in simultaneous transformants with T21, T67, and T831011213 (FfDTFUM21_6_13 in Figure 3 and Table 2). These results suggested that causative mutations of fumonisin nonproduction in G1 strain are distributed in T21, T67, and T831011213 regions. In order to narrow the regions of mutation(s), simultaneous transformations in T21 and T67 regions were conducted in G1 strain. Although fumonisin production had not been recovered with either of T21 or T67 (FfT21FUMKOD and FfT67FUMKOD in Figure 3), it was recovered with the simultaneous transformations with these (FfT67T21FUMKOD2 and FfDT21T67KOD in Figure 3). 

To specify the location of the causative mutation(s) in T67 region, plasmid carrying *FUM6* (pBSNT67046T-3 in Table S3) or *FUM7* (pBSNT67141A-3 in Table S3) was created by frameshift of either of *FUM6* or *FUM7* in the plasmid carrying T67 fragment (pCBT67KOD-1) (Table S3) and transformed into FfT21FUMKOD (#2), which is a transformant with T21 (FfT6TT21FUMKOD2 and FfT7AT21FUMKOD2 in Figure 3). Fumonisin production was recovered in FfT7AT21FUMKOD2 transformants (Figure 3 and Table 2). These results suggested that mutations causing fumonisin nonproduction in G1 strain were distributed in *FUM21* and *FUM7*. Sequence comparison between G1 and G9 strain indicated that three amino acid substitutions are present in FUM7 (Figure S3) and two nucleotide substitutions in the intergenic region (possible bidirectional promoter region) between *FUM6* and *FUM7* (Figure S4). 

### 2.5. Causative Mutation in FUM21

It was assumed that the causative mutation of *FUM21* in G1 strain is either that of g.888G>C (p.D261H) or g.2551G>T (p.G678*). In order to identify it, the transformation plasmid that carries a point mutation at g.888G>C (p.D261H) or g.2551G>T (p.G678*) in *FUM21* of G9 strain was created (pDT21G888C-1 and pDT21G2551T-2 in Table S3). Each plasmid was transformed into FfT67831011213 (#30), which is a simultaneous transformant with T67 and T831011213. The created transformants were FfDTFUM21G888C-6-13 and FfDTFUM21G2551T-6-13 (Figure 3). Fumonisin production was not recovered in all 22 FfDTFUM21G2551T-6-13 transformants (Figure 3 and Table 2), while it was recovered in five out of 18 FfDTFUM21G888C-6-13 transformants (Figure 3 and Table 2). Failure of fumonisin production recovery with pDT21G2551T may attribute partial integration of T21 rather than g.2551G>T substitution. Therefore, PCR detection of T21 was conducted and full-length integration was confirmed in 13 out of 22 FfDTFUM21G2551T-6-13 transformants and five out of 18 FfDTFUM21G888C-6-13 transformants. Expression of *FUM21* and *FUM8* in a representative transformant were investigated. *FUM8* was not expressed in FfDTFUM21G2551T-6-13(#1), whereas it was expressed in FfDTFUM21G888C-6-13(#5). These results suggested that g.2551G>T (p.G678*) (Figure 2) in *FUM21* is the cause of fumonisin nonproduction though additional mutation(s) is/are present in T7 regions in G1 strain.

We sequenced the terminal portion of *FUM21* in an additional three fumonisin nonproducing strains (GL-24, Gfc0625008 and Gfc1034001) and three fumonisin producing strains (Gfc0821004, Gfc0009063 and 41-79) that were used in the previous study [17] to investigate the g.2551G>T. Three fumonisin nonproducing strains had T as G1 strain while three fumonisin producing strains had G at the site (Table S4). We also checked the g.2551G>T in nine *F. fujikuroi* strains, the whole genome sequences that was investigated [20]. One of the nine strains (B14) is an apparent fumonisin producer, and the other strains are fumonisin non- or low-producers [20]. B14 strain has G at the site. In case of fumonisin non or low producers, four strains have T and four strains including IMI58289 have G at the site (Table S4).

## 3. Discussion

A precise understanding of mycotoxin producibility of fungal species is important to clarify its mycotoxin contamination risks. Fumonisin is one of the most potent mycotoxins responsible for food safety. Rice is major grain providing food for the world population and, therefore, fumonisin producibility of rice infecting fungus, *F. fujikuroi*, is a crucial risk. In order to scrutinize fumonisin producibility of *F. fujikuroi* G-group strains at the gene level, the causative mutations of fumonisin nonproduction in G1 strain was clarified in this study. 

G1 strain carries a FUM cluster as fumonisin producing G9 strain, though *FUM17* is a pseudo gene in both G1 and G9 strains, as it was previously reported in the *F. fujikuroi* strain IMI58289 [33]. *FUM16* may complement *FUM17* function because both genes have sequence similarity to tomato longevity assurance factor (ASC-1) of *Alternaria alternata* f. sp *lycopersici* and *FUM16* disruption did not affect fumonisin producibility in *F. verticillioides* [28]. High-sequence homology was observed in *FUM* genes between G1 and G9 strains (Table 1), such as an aflatoxin biosynthetic gene cluster between aflatoxin producers *Aspergillus parasiticus* or *Aspergillus flavus* and nonproducer *Aspergillus oryzae* RIB40 [34]. Among 16 *FUM* genes, a comparatively higher number of amino acid substitutions were observed in *FUM16*, *FUM18* and *FUM19* (Table 1) which are known to be not essential for fumonisin production by gene disruption studies [28,29]. In consideration of co-localization of these *FUM* genes in the FUM cluster, the origin of essential *FUM* gene regions and non-essential may be different. 

The dysfunction of a transcription factor is one of the possible mechanisms of mycotoxin nonproduction [30]. Pre-termination of ORF was observed in the transcription factor (*aflR* gene) of *Aspergillus sojae*. This mutation causes aflatoxin nonproduction by the dysfunction of the protein with the deletion of 62 amino acids in the respective gene product [35]. *FUM21* encodes a positive transcriptional regulator of other *FUM* genes. FUM21 is a GAL4 type zinc finger protein [29], a C-terminal region that is thought to be critical for transcriptional activation [36]. The g.2551G>T (p.G678*) in *FUM21* was identified as one of the causative mutations of fumonisin nonproduction in G1 strain. This mutation lacks 11 amino acids at C-terminal of FUM21 and, presumably, it is dysfunctional because positive conversion of *FUM6*, *FUM8*, and *FUM10* expressions was observed in *FUM21* complemented transformant (FfT21FUMKOD (#2) in Figure 1). 

*F. fujikuroi* IMI58289 strain is a fumonisin low producer [18,20]. This strain does not have the g.2551G>T (p.G678*) substitution in *FUM21* (FFUJ-09241) and, therefore, retains the normal C-terminal of FUM21. Instead, the low level of *FUM21* expression was revealed as only the cause of fumonisin low-production in IMI58289 strain [18]. On the other hand, failure of fumonisin production recovery by *FUM21* complementation in G1 strain suggested that additional cause of fumonisin nonproduction is present in this strain. The successful fumonisin production recovery by the simultaneous complementation of *FUM21* and *FUM7* indicated that the additional mutation(s) is present in *FUM7* encoding alcohol dehydrogenases. Similarly, multiple mutations in the biosynthetic gene cluster of secondary metabolites were reported on aflatoxin and gibberellin nonproduction in *A. oryzae* and *F. proliferatum*, respectively [34,37,38]. Comparing to G9 strain, three amino acid substitutions in FUM7 and 2 nucleotide substitutions in the putative bidirectional promoter between *FUM6* and *FUM7* were detected in G1 strain (Figures S3 and S4). These substitutions might be the cause of the dysfunctionality of *FUM7* in G1 strain. 

We detected the mutations in *FUM21* and *FUM7* that affect fumonisin production in G1 strain but further mutation(s) could be present in the FUM cluster of G1 because the level of fumonisin production recovery in FfDT21T67FUMKOD, FfDT67T21FUMKOD2, and FfT7AT21FUMKOD2 (Table 2) were greatly lower than FfDTFUM21_6_13. 

We investigated the cause of fumonisin nonproduction in G1 strain. However, the causative mutation of fumonisin nonproduction may be varied in *F*. *fujikuroi.* A partial deletion of FUM cluster was reported in *F. fujikuroi* strain FGSC8932, though its fumonisin production has not been investigated [32]. Suga et al. [19] failed PCR amplification of a region in *FUM18* in three out of the five G-group strains and, therefore, this region may be absent in these strains. Further study would reveal the variation of causative mutations of fumonisin nonproduction in *F. fujikuroi*. 

## 4. Materials and Methods

### 4.1. Fungal Strains

*Fusarium fujikuroi* strains, fumonisin production of that was investigated in the previous report were used [17]. G9 from maize, Gfc0821004 from rice seed, Gfc0009063 from strawberry, and 41-79 from wheat were fumonisin producers and G1, Gfc0625008, Gfc1034001, and GL24, were from rice and were fumonisin nonproducers. GL24 and 41-79 were from the US and the remaining strains were from Japan.

### 4.2. Crossing

Five pieces of 5 cm rice straw were put in a 200 mL beaker with a lid of aluminum foil and sterilized by autoclave. A fumonisin producer G9 (MAT1-1 type) and a nonproducer G1 (MAT1-2 type) were transplanted to the center of a piece and incubated for one week at 25 °C with a lid of aluminum foil/parafilm under the mixture light of black and white fluorescent. Shallow sterile water was poured into a beaker and was removed the next day. The perithecia that developed in two weeks was thoroughly rinsed by sterile water. A cover glass was placed on a perithecium on a glass slide and the perithecium was crushed with a little pressure. Ascospores were collected by several hundred μL of sterile water and spread to a minimal medium containing 0.05% (vol/vol) tergitol type NP-10 and 2% (wt/vol) L-sorbose instead of 3% sucrose (MMTS medium) [39]. Progenies were maintained on potato dextrose agar (PDA) after single-spore isolation and kept at 6 °C for short-term storage and at −80 °C in 50% glycerol for long-term storage. 

### 4.3. DNA Extraction

Genomic DNA was extracted from 3–4 day old mycelium (2–3 cm diameter) cultured on potato dextrose broth (PDB) by using potassium ethyl xanthogenate solution, as previously described [40]. The final DNA pellet was dissolved in 200 μL of water, and 5 ng/μL was used for PCR. 

### 4.4. SNP Analysis by Luminex and MAT Typing

A multiplex PCR was performed to prepare template DNA for allele-specific primer extension (ASPE) reactions. The PCR for *TEF* and *FUM* amplification was set up with 0.025 units rTaq polymerase (Takara Bio Inc., Otsu, Japan) in a 10 μL reaction mixture, containing 1× reaction buffer, 200 μM dNTPs, 1 μM of each HS438 and HS439 primer [17] (Table S5), and 0.5 μM of each HS398, HS399, HS506, and HS519 primer (Table S5) and 5 ng genomic DNA. The PCR for *CPR* and *P450-4* amplification were similarly setup but contained 1 μM of each P138-5, HS556, HSP450-4-GD1, and P450-4-GD2 primer [19,37,41] (Table S5). PCR was performed in an iCycler thermal cycler (Bio-Rad Laboratories), using the following cycling parameters: 94 °C for 2 min, 35 cycles of 94 °C for 1 min, 58 °C for 1 min, and 72 °C for 1 min. Each 2.5 μL PCR mixture of *TEF*/*FUM*, and *CPR*/*P450-4*, 0.2 μL ExoSAP-IT (GE Healthcare Life Sciences, Uppsala, Sweden), and 1.8 μL water were mixed and kept at 37 °C for 30 min and then 80 °C for 15 min. ASPE reactions were performed according to the manufacturer’s instructions using Platinum Genotype Tsp DNA Polymerase (Invitrogen Life Technologies, Carlsbad, CA, USA), HS641, HS642, HS540, HS541, HS542, HS543, HS557, HS558, HS559, and HS560 primer (Table S5) in 20 μL volume but with extension step at 50 °C for 1 min. Biotin labelled products were sorted by hybridization with polystyrene microspheres coated with anti-tag sequences: LUA-12, -65, -67, -76, -87, -97, -16, -28, -2, and -14. Hybridizations were performed in 45 μL volumes with 1×Tm hybridization buffer (0.2 M NaCl, 0.1 M Tris, 0.08% Triton X-100), along with pH 8.0 including 20 μL of extension product. The samples were incubated for 90 s at 96 °C, followed by 30 min at 37 °C, and then transferred to a 96-well filtration plate (Multi Screen HTS; Millipore Corp., Billerica, MA, USA). Liquid was removed by a vacuum manifold and then adding 100 μL of 1×Tm hybridization buffer was repeated twice and again liquid was removed by a vacuum manifold. Microspheres were resuspended in a 100 μL of 1×Tm hybridization buffer containing 2 μg/mL streptavidin-R-phycoerythin (Invitrogen Life Technologies). The median fluorescence intensity (MFI) of 100 microspheres was measured with a Luminex 100 flow cytometer (Luminex Corporation, Austin, TX, USA) after incubation for 15 min at 37 °C. More than 100 MFI value after subtraction of background of MFI value obtained by water instead of extension product was used for SNP determination. SNP was determined based on the ratio with more than twice the MFI value between the paired microspheres corresponding to SNP: LUA-12/-65, -67/-76, -87/-97, -16/-28, and -2/-14.

The mating type was determined by PCR with the primer fusALPHAfor and fusALPHArev for MAT1-1, and fusHMGfor and fusHMGrev for MAT1-2 [42].

### 4.5. PCR and Sequencing

PCR was performed by either an iCycler or T100 thermal cycler (Bio-Rad Laboratories Hercules, CA, USA). A to J regions in a FUM cluster of G9 strain (Figure S1) was amplified by PCR using AccuTaq LA DNA Polymerase (Sigma, St Louis, MO, USA) with the following cycling parameters: 94 °C for 30 s, 30 cycles of 94 °C for 15 s, annealing temperature for 20 s, and 68 °C for 8 min. Annealing temperature was 48 °C for A, E, F, and J regions and 56 °C for B, C, D, G, H, and I regions. The primer pairs were shown in Table S6. Flanking region (Figure S1) was amplified by dual-suppression-PCR with the primer shown in Table S6 [43] because the whole genome sequence of *F. fujikuroi* had not been published at that time [33]. Genomic DNA digested with each of the blunt-end restriction endonucleases *Hae* III, *Eco*R V, and *Alu* V (New England Biolabs, Beverly, MA, USA) were used for dual-suppression-PCR because their recognition sites are not present in the nucleotide sequence between IP1 primer (Table S6) and the known terminal nucleotide. DNA Ligation Kit Ver. 2 (Takara) was used for the ligation of the adaptor that prepared with HS470 and HS471 (Table S6) to the genomic DNA digested by the restriction endonuclease. IP1/AP1 primers were used for the first PCR and IP2/AP2 primers were used for the nested PCR by AccuTaq LA DNA Polymerase (Sigma). Ca. 1000 bp-DNA was amplified from *Eco*R V digested genomic DNA and it was used for direct sequencing with IP2 and AP2 primer (Table S6). K to R regions in a FUM cluster of G1 strain (Figure S1) was amplified by PCR with the same conditions except for annealing temperature at 52 °C and 68 °C for 10 min. PCR products were directly sequenced as previously described [40]. Big Dye Terminator V3.1 with cycle sequencing kits (Life Technologies) using the primers shown in Table S6, and the sequence was obtained by an ABI 3100 genetic analyzer (Life Technologies). PCR product of A region amplified with HS399 and HS487 primer was failed to be sequenced by these primers and it was assumed that PCR product had HS399 sequence at both terminals. Then, PCR product was cloned into pCR4-TOPO (Invitrogen) and terminal regions were sequenced with M13M4 and M13RV primer (Table S6). Nucleotide sequences data was processed with Chromas Pro (Technelysium Pty., Tewantin, Queensland, Australia) and Genetyx (Genetyx, Tokyo, Japan). Sequences of FUM cluster of G9 and G1 strains were available in DDBJ/EMBL/GenBank database with accession numbers HQ622717 and JN807324, respectively. 

An SNP at 2551st in *FUM21* of the strains except for G9 and G1 was determined by direct sequencing of the PCR products amplified using the HS744 primer (5′-ATACTGCTGCCATTACGCAA-3′) and HS561 (Table S6). PCR was performed using KOD-Plus-Neo DNA polymerase (Toyobo, Tokyo, Japan) with the following cycling parameters: 94 °C for 2 min, 30 cycles of 98 °C for 10 s, 61 °C for 20 s, and 68 °C for 3 min. The SNP of *F. fujikuroi* strains in [20] were obtained from the BioProject PRJEB14872 (ID: 412609) at the National Center for Biotechnology Information (NCBI).

Integration of T21 fragment into the transformants was confirmed by PCR of 6 kbp-DNA using Accu Taq LA DNA Polymerase (Sigma), M13M4 primer and HS685 (5′-ATGCGGCCGCTCCTCCACCAGATGATGACA-3′) with the following cycling parameters: 94 °C for 30 s, 30 cycles of 94 °C for 15 s, 54 °C for 20 s, and 68 °C for 7 min. 

### 4.6. Construction of the Transformation Vector

A part of a FUM cluster was amplified from G9 strain by PCR using the primer pairs shown in Table S3. PCR products treated with *Not* I (New England Biolabs) were inserted into the *Not* I site in pCB1004 [44], pDNAT1 [45] or pBSNII99-3 to create transformation vectors using DNA Ligation Kit Ver. 2 (Takara) (Table S3). pBSNII99-3 was created by the insertion of the geneticin resistance cassette that was cut out with *Eco* RV from pBS99 into *Nae* I site of pBluescript II KS (+) (Agilent Technologies). pBS99 was created by the geneticin resistance cassette from pII99 [46]. The geneticin resistance cassette in pII99 was cut out with *Bgl* II and *Xba* I though *Xba* I terminal was blunted before *Bgl* II digestion. pBS99 was created by the insertion of this geneticin resistance cassette at *Bam* HI and *Sma* I site of pBluescript II KS (+). Inserted DNA in the plasmid except for pDT21-1 was confirmed by sequencing with M13M4 and M13RV primers. Inserted DNA in pDT21-1 was confirmed by sequencing with HS743 (5′-TCTACATGAGCATGCCCTGCCCCTGAGGGCCC-3′) and M13M4 primers. pDT21G888C-1 with G to C substitution at the 888th nucleotide in *FUM21* and pDT21G2551T-2 with G to T substitution at the 2551st nucleotide in *FUM21* were created from pDT21-1 by QuickChange II XL Site-Directed Mutagenesis Kit (Stratagene, La Jolla, CA, USA) with HS748/HS749 primer and HS746/HS477 primer, respectively (Table S3). The nucleotide substitution in pDT21G888C-1 was confirmed by sequencing with HS744 primer (5′-ATACTGCTGCCATTACGCAA-3′). The nucleotide sequence of inserted T21 including the nucleotide substitution in pDT21G2551T-2 was confirmed by sequencing with primer HS608, HS618, HS622, HS617, HS607, HS599, HS585, HS489, HS561, HS562, M13M4 (Table S6), and HS743. 

Dysfunction of *FUM7* or *FUM6* in pBSNT67-1 was attained by the frameshift with a nucleotide insertion. pBSNT67046T-3 with T insertion between 46/47th nucleotide in *FUM7* and pBSNT67141A-3 with A insertion between 141/142th nucleotide in *FUM6* were created from pBSNT67-1 by QuickChange II XL Site-Directed Mutagenesis Kit (Stratagene) with HS949/HS950 primer and HS947/HS948 primer, respectively (Table S3). The nucleotide insertion in pBSNT67046T-3 and pBSNT67141A-3 was confirmed by sequencing with HS533 and HS494 primer, respectively (Table S6). 

### 4.7. Fungal Transformation

The fungal transformation was performed according to [47], but PDB instead of mung bean liquid media was used for producing budding cells. One percent water agar containing 250 μg of hygromycin B (Wako, Osaka, Japan)/mL, 800 μg of nourseothricin (Axxora, San Diego, CA, USA)/mL, or 450 μg of geneticin (G418 disulfate salt, Sigma)/mL was used for selection of transformant-colonies. The transformants created in this study were shown in Table S7. 

### 4.8. RT-PCR

Total RNA was extracted from mycelium cultured in GYAM (8.0 mM L-asparagine, 1.7 mM NaCl, 4.4 mM K_2_HPO_4_, 2.0 mM MgSO_4_, 8.8 mM CaCl_2_, 0.05% yeast extract, 0.24 M glucose, 5.0 mM malic acid) [27,48] for seven days. The Maxwell RSC and The Simply RNA Tissue kit (Promega, Madison, WI, USA) were used for RNA extraction. RT-PCR was performed with a Titan One Tube RT-PCR Kit (Roche Diagnostics, Mannheim, Germany) with the primer pair shown in Table S2 according to the manufacturer’s instructions. PCR except for *FUM8* was performed in a T100 thermal cycler (Bio-Rad) using the following cycling parameters: 45 °C for 30 min and 94 °C for 2 min, 30 cycles of 94 °C for 10 s, 56 °C for 30 s, and 68 °C for 1 min. In the case of *FUM8*, the cycle number was 35 and the annealing temperature was 51 °C instead of 58 °C.

### 4.9. Fumonisin Analysis

Strains or transformants were cultured on a 5 g cracked maize seed which included 2 mL of water at 25 °C for 10 days. Fumonisins were extracted with 25 mL of methanol/water (3:1, *v*/*v*) by reciprocal shaking for 30 min. One hundred μL of extract was used for a competitive enzyme immunoassay, RIDASCREEN^®^FAST Fumonisin kit (R-Biopharm, Darmstadt, Germany) after dilution 14 times with water according to the manufacturer’s instructions. Fumonisin concentration higher than 0.222 μg/gram was considered as fumonisin production positive. In case the transformant with fumonisin production positive was detected, fumonisins in the extract of three individual transformants for a transformation plasmid were analyzed by LC-MS/MS according to [19]. The experimental culture was repeated in triplicate. FB_1_ (Enzo Life Sciences, Lausen, Switzerland), FB_2_ (Enzo Life Sciences), and FB_3_ (Iris Biotech, Marktredwitz, Germany) dissolved in acetonitrile/water (1:1, *v*/*v*) and were standard solutions. Working solutions containing FB_1_, FB_2_, and FB_3_ at concentrations between 0.05–5.0 mg/L (FB_1_/FB_2_: 0.1, 0.2, 0.5, 1.0, 2.0, 5.0 mg/L, FB_3_: 0.05, 0.1, 0.2, 0.4, 0.6, 1.0 mg/L, respectively, in six bottles) were used to create a calibration curve. The concentration (X) (FB_1_, FB_2_, and FB_3_) and corresponding peak area ratio were plotted for the 6 bottles of the working solution. The limits of qualification (LOQs) for the fumonisin analysis was the lowest concentration (FB_1_, FB_2_: 0.1 mg/L, FB_3_: 0.05 mg/L) on the calibration curves for FB_1_, FB_2_, and FB_3_. In cases where the concentration of the sample exceeded the range given by the calibration curve, the sample was diluted 10 times and reanalyzed.

## Figures and Tables

**Table 1:** Sequence homology of FUM gene between a fumonisin producing strain Gfc0825009 and a nonproducing strain Gfc0801001.

Gene	Gene ID ^a^	Predictive Function ^b^	Homology (%)		Length of Amino Acid ^c^	The Number of Different Amino Acid ^d^
			Nucleic Acid	Amino Acid		
*FUM21*	FFUJ_09240 ^e^	Zn(II)2Cys6 type-transcriptional regulator	99.6	99.8	688 ^e^	1 substitution, 11 deletion
*FUM1*	FFUJ_09241	Polyketide synthase	99.6	99.6	2580	8 substitutions
*FUM6*	FFUJ_09242	Fumonisin oxygenase	99.3	99.2	1115	8 substitutions
*FUM7*	FFUJ_09243	Alcohol dehydrogenase	98.8	99.2	420	3 substitutions
*FUM8*	FFUJ_09244	Aminotransferase	98.2	98.3	830	14 substitutions, 2 insertion
*FUM3*	FFUJ_09245	Fumonisin 5-oxygenase	99.3	99.6	300	1 substitution
*FUM10*	FFUJ_09246	Tricarballylic esterification	99.5	99.4	561	3 substitutions
*FUM11*	FFUJ_09247	Tricarballylic esterification	98.7	98.3	300	5 substitutions
*FUM2*	FFUJ_09248	Fumonisin 10-oxygenase	98.8	99.0	502	5 substitutions
*FUM13*	FFUJ_09249	C3 carbonyl reductase	99.5	99.5	367	2 substitutions
*FUM14*	FFUJ_09250	Tricarballylic esterification	99.1	99.1	556	5 substitutions, 5 insertion
*FUM15*	FFUJ_09251	Cytochromosome P450 monooxygenase	98.7	98.6	599	8 substitutions
*FUM16*	FFUJ_09252	Fatty acyl-coenzyme A synthetase	98.4	98.7	682	9 substitutions, 10 deletion
*FUM17*	- ^f^	Similarity to tomato longevity assurance factor (ASC-1) of *Alternaria alternata* f. sp. *lycopersici*	- ^f^	- ^f^	- ^f^	- ^f^
*FUM18*	FFUJ_09253	Similarity to tomato longevity assurance factor (ASC-1) of *Alternaria alternata* f. sp. *lycopersici*	99.1	97.5	413	10 substitutions
*FUM19*	FFUJ_09254	Similarity to ATP-binding cassette (ABC) multidrug resistant transporter	99.1	99.0	1505	15 substitutions

^a^: Gene ID of whole genome sequence of the strain IMI58289 [33]. ^b^: Predictive function of *FUM* genes, except for *FUM21* [30], was referred to [29]. ^c^: Value of Gfc0825009. ^d^: Sequence of Gfc0801001 compared to that of Gfc0825009. ^e^: Intron region was modified from that of IMI58289 [33] in the database according to *FUM21* (FVEG_14633, protein ID: XP_018742371.1) of *Fusarium verticillioides*. ^f^: *FUM17* of both Gfc0825009 and Gfc0801001 are pseudogene.

**Table 2:** Fumonisin production of transformants ^a^.

Transformant ^b^	First Time Culture			Second Time Culture			Third Time Culture		
	FB_1_	FB_2_	FB_3_	FB_1_	FB_2_	FB_3_	FB_1_	FB_2_	FB_3_
FfDTFUM21_6_13(#1)	43.63	1.12	>10.00	>50.00	7.57	7.96	4.62	0.85	1.29
FfDTFUM21_6_13(#2)	46.56	1.86	>10.00	>50.00	5.76	>10.00	>50.00	3.73	7.78
FfDTFUM21_6_13(#3)	24.80	1.01	8.35	0.41	ND	0.12	0.20	ND	0.12
FfDTFUM21G888C_6_13(#5)	3.70	0.20	>1.00	47.03	4.09	>10.00	27.15	2.45	5.22
FfDTFUM21G888C_6_13(#6)	37.23	1.28	>10.00	ND	ND	ND	ND	ND	ND
FfDTFUM21G888C_6_13(#7)	>50.00	2.39	>10.00	0.19	ND	0.08	ND	ND	ND
FfDT21T67FUMKOD(#11)	1.57	0.26	0.98	0.95	0.19	0.30	1.33	0.24	0.36
FfDT21T67FUMKOD(#16)	3.62	0.38	0.71	2.88	0.93	0.94	1.72	0.67	0.79
FfDT21T67FUMKOD(#17)	0.57	0.17	0.09	2.59	1.42	0.36	0.14	ND	ND
FfT67T21FUMKOD2(#7)	0.20	ND	0.06	ND	ND	ND	ND	ND	ND
FfT67T21FUMKOD2(#11)	ND	ND	ND	ND	ND	ND	ND	ND	ND
FfT67T21FUMKOD2(#16)	ND	ND	ND	ND	ND	ND	ND	ND	ND
FfT7AT21FUMKOD2(#23)	0.19	ND	0.15	ND	ND	ND	ND	ND	ND
FfT7AT21FUMKOD2(#34)	0.11	ND	0.10	ND	ND	ND	ND	ND	ND
FfT7AT21FUMKOD2(#18)	0.13	ND	0.08	1.06	ND	0.25	0.48	ND	0.21
Gfc0825009(Donor strain)	>50.00	>50.00	3.70	44.40	35.70	2.13	42.60	36.66	2.59
Gfc0801001(Original strain)	ND	ND	ND	ND	ND	ND	ND	ND	ND

^a^: Fumonisins from a part of the transformants with ELISA positive in three-time culture were quantified by liquid chromatography-tandem mass spectrometry (LC- MS/MS). The limit of quantification (LOQ) is 0.1 ppm for FB_1_, FB_2_, and 0.05 ppm for FB_3_. ND means less than LOQ. ^b^: (#) indicates individual transformant number.

## Supplementary Materials

The following are available online at https://www.mdpi.com/2072-6651/11/4/200/s1, Figure S1: PCR amplification for sequencing of FUM cluster, Figure S2: PCR detection of T21 in the transformant, Figure S3: Comparison of the amino acid sequences of FUM7 between fumonisin producing strain Gfc0825009 (upper) and fumonisin non-producing strain Gfc0801001 (lower), Figure S4: Comparison of nucleotide sequences of putative bidirectional promoter region of *FUM6* and *FUM7* between fumonisin producing strain Gfc0825009 (upper) and fumonisin non-producing strain Gfc0801001 (lower), Table S1: Result of SNP analyses and fumonisin production of the progenies between Gfc0825009 and Gfc0801001, Table S2: Primer used for RT-PCR, Table S3: Plasmid created in this study, Table S4: SNP at 2551st in *FUM21*,Table S5: Primer used for Luminex assay, Table S6: Primer used for sequencing of FUM cluster, Table S7: Transformants created in this study.
