# Supplementary material for: A Natural Variation of Fumonisin Gene Cluster Associated with Fumonisin Production Difference in Fusarium fujikuroi
Source: Toxins (Basel). 2019 Apr 3;11(4):200. doi: 10.3390/toxins11040200 (PMC6521250; doi:10.3390/toxins11040200)
Supplement: Supplementary file 1 [file toxins-11-00200-s001.pdf]

Supplementary Materials: A Natural Variation of Fumonisin Gene Cluster Associated with Fumonisin Production Difference in *Fusarium fujikuroi*

Sharmin Sultana, Miha Kitajima, Hironori Kobayashi, Hiroyuki Nakagawa, Masafumi Shimizu, Koji Kageyama and Haruhisa Suga

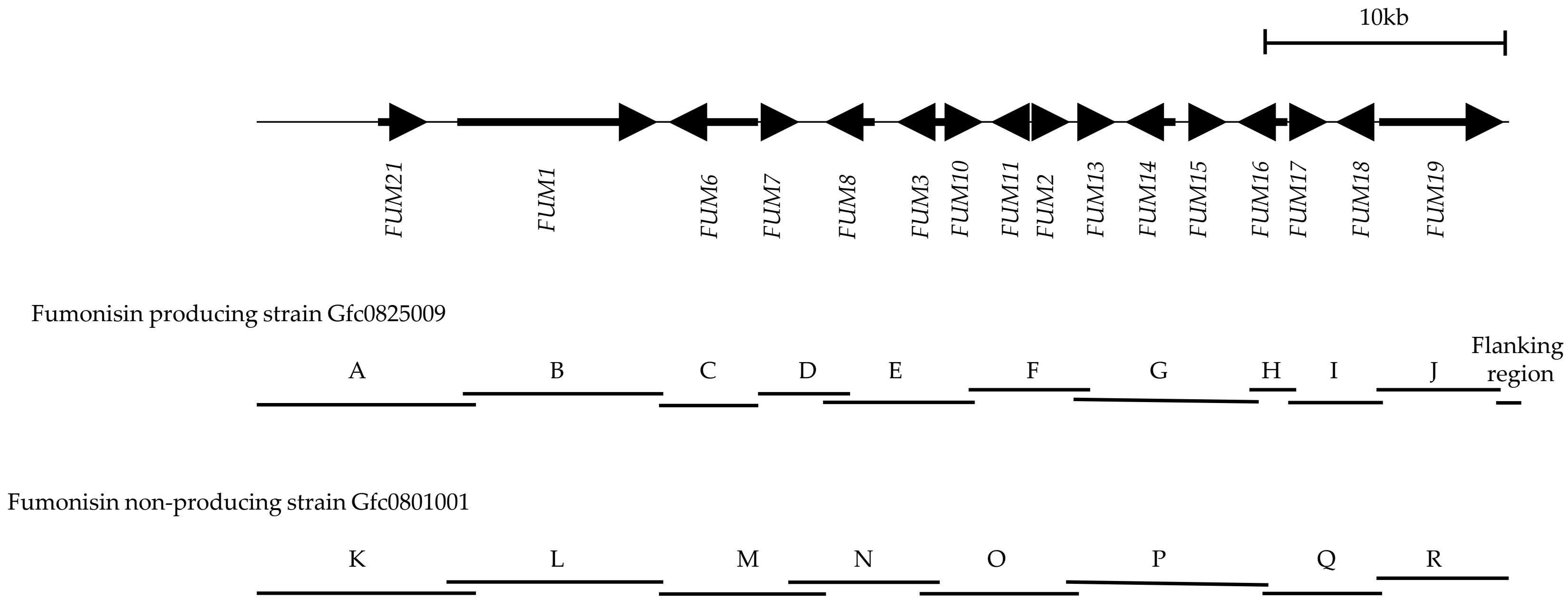

Figure S1. PCR amplification for sequencing of FUM cluster.

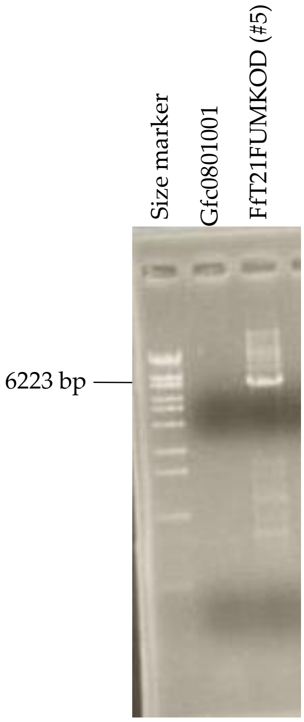

Figure S2. PCR detection of T21 in the transformant. Here Gfc0801001 was used as negative control. The target amplification size was 6 kbp.

MSSLDHHHVHPANSRNPKPCVSYGIPFWEACTRHAKAFNSTRIYIVSSRSLSKSQALQDLKTSLGLFRIAGEYNGITQHT 80
MSSLDHHHVHPANSRNPKPCVSYGIPFWEACTRHAKAFNSTRIYIVSSRSLSKSQALQDLKTSLGLFRIAGEYNGITQHT 80

AWDQVFGLVHDLKETRADLIITLGGGSVTDGVKLARLLVANNIMALEQAESLLSRCEPGKPSDE**G**VKPASIPVINVPTTL 160
AWDQVFGLVHDLKETRADLIITLGGGSVTDGVKLARLLVANNIMALEQAESLLSRCEPGKPSDE**S**VKPASIPVINVPTTL 160

SGAEFTRAAGATYTESNHKKRIIIHQSMYADFVVLDPELSLTTPARYWISTGIRAVDHFVEGIYGNMSMVRGDTSGSDQF 240
SGAEFTRAAGATYTESNHKKRIIIHQSMYADFVVLDPELSLTTPARYWISTGIRAVDHFVEGIYGNMSMVRGDTSGSDQF 240

IEKDIQASLADLLVALLQTVDDWHNHDARLRQLLALKDCPRAGHNGVGASHGIGHQLGPFGVGHGETSCIILPCVLKYNW 320
IEKDIQASLADLLVALLQTVDDWHNHDARLRQLLALKDCPRAGHNGVGASHGIGHQLGPFGVGHGETSCIILPCVLKYNW 320

S**N**GDARLRSKLQLIMDVFWGNAVLTKLLLLRGLRPQDADPGDVLAAYISALGMPNSLGKYGINQDKFHQIADNAMEDVCT 400
S**K**GDARLRSKLQLIMDVFWGNAVLTKLLLLRGLRPQDADPGDVLAAYISALGMPNSLGKYGINQDKFHQIADNAMEDVCT 400

QLNPV**V**LDKDRVVEILYMAA* 420
QLNPV**A**LDKDRVVEILYMAA* 420

Figure S3. Comparison of the amino acid sequences of FUM7 between fumonisin producing strain Gfc0825009 (upper) and fumonisin non-producing strain Gfc0801001 (lower). Amino acid substitutions were indicated by red letters.

```
GAAGCTAGTTGAAGTCGGCGCAAAACAAACTGTCAAGGTAGGGTGGTGGTTGAAGGGCCGTTTGGGTTGGGGCTTGTTGA  80
GAAGCTAGTTGAAGTCGGCGCAAAACAAACTGTCAAGGTAGGGTGGTGGTTGAAGGGCCGTTTGGGTTGGGGCTTGTTGA  80

TTTATCGTACATAGTTCTTCTATGTCATCCATGATTAATAGGCAATTACTGCGGTTTTATTATACTCTGCACCACATACG  160
TTTATCGTACATAGTTCTTCTATGTCATCCATGATTAATAGGCAATTACTGCGGTTTTATTATACTCTGCACCACATACG  160

ACTACAAACTAGTTGGATGGCTACCCTCACAGAGGGACTTTGCGCACCGCCTCTAGGTTCCCTAACATGCCCAACCAGGC  240
ACTACAAACTAGTTGGATGGCTACCCTCACAGAGGGACTTTGCGCACCGCCTCTAGGTTCCCTAACATGCCCAACCAGGC  240

CAGCACGTGCAGCGTTAACGGAAACAAGACTCGTCGCGGGTATGTAGTAGACCGGAAGACAAAGGGCGATTTTCGGCTGC  320
CAGCACGTGCAGCGTTAACGGAAACAAGACTCATCGCGGGTATGTAGTAGACCGGAAGACAAAGGGCGATTTTCGGCTGC  320

GGATCTACAAAAGCGGCAAACCTTCCGCTCGGCTACTGAAATGGGAATTGAGTGGCGGTAAAGAGCCGACAGTCCCAGAG  400
GGATCTACAAAAGCGGCAAACCTTCCGCTCGGCTACTGAAATGGGAATTGAGTGGCGGTAAAGAGCCGACAGTCCCAGAG  400

AGCTGAAAGACTGGTCTCAAATTAGAAAAGGATGTTGAATTTCAACATCAAGACAATTTTTATTTTACGCGGTGCACGCT  480
AGCTGAAAGACTGGTCTCAAATTAGAAAAGGATGTTGAATTTCAACATCAAGACAATTTTTATTATACGCGGTGCACGCT  480

GTCTAGCACCGTTGATAGGCCTTCACATTAGATCAAGCCAAGCAAAAGTGGAC                            533
GTCTAGCACCGTTGATAGGCCTTCACATTAGATCAAGCCAAGCAAAAGTGGAC                            533
```

Figure S4. Comparison of nucleotide sequences of putative bidirectional promoter region of *FUM6* and *FUM7* between fumonisin producing strain Gfc0825009 (upper) and fumonisin non-producing strain Gfc0801001 (lower). Nucleotide substitutions were indicated by red letters.

Table S1. Result of SNP analyses and fumonisin production of the progenies between Gfc0825009 and Gfc0801001

Progeny	SNP data[a]					MAT type[b]	Fumonisin (ppm)[c]
	TEF_T618G	CPR_C1152A	P4504_C842T	FUM1_G423A	FUM18_G51T		
Gfc0825009 (parent)	T	C	C	G	G	1-1	5.7
Gfc0801001 (parent)	G	A	T	A	T	1-2	ND
Gfc①CP91002	G	A	C	G	G	1-2	>6.0
Gfc①CP91005	T	C	C	A	T	1-1	ND
Gfc①CP91007	T	C	T	A	T	1-1	ND
Gfc①CP91008	G	A	C	A	T	1-2	ND
Gfc①CP91009	G	A	T	G	G	1-1	3.6
Gfc①CP91011	T	C	T	G	G	1-1	>6.0
Gfc①CP91017	T	C	T	G	G	1-1	4.2
Gfc①CP91019	T	A	T	A	T	1-1	ND
Gfc①CP91020	G	C	C	G	G	1-2	5.2
Gfc①CP91022	T	C	C	A	T	1-1	ND
Gfc①CP91023	T	A	C	G	G	1-1	1.6
Gfc①CP91024	T	A	T	G	G	1-2	>6.0
Gfc①CP91027	T	C	T	A	T	1-1	ND
Gfc①CP91029	T	C	T	G	G	1-1	5.3
Gfc①CP91033	T	A	T	G	G	1-1	4.8
Gfc①CP91034	G	C	C	A	T	1-2	ND
Gfc①CP91035	G	C	T	G	G	1-2	3.9
Gfc①CP91041	G	A	T	A	T	1-2	ND
Gfc①CP91045	G	C	C	A	T	1-2	ND
Gfc①CP91049	G	A	C	G	G	1-2	>6.0
Gfc①CP91051	T	A	T	A	T	1-1	ND
Gfc①CP91053	T	A	T	G	G	1-2	>6.0
Gfc①CP91054	G	C	C	G	G	1-1	3.7
Gfc①CP91055	G	C	T	G	G	1-2	5.4
Gfc①CP91059	G	C	C	A	T	1-2	ND
Gfc①CP91062	G	A	C	A	T	1-2	ND
Gfc①CP91063	T	C	T	A	T	1-1	ND
Gfc①CP91065	T	C	T	A	T	1-1	ND
Gfc①CP91066	T	C	C	A	T	1-1	ND
Gfc①CP91067	T	C	C	A	T	1-1	ND
Gfc①CP91068	T	A	T	A	T	1-1	ND
Gfc①CP91070	T	A	C	G	G	1-1	1.9
Gfc①CP91071	T	A	C	G	G	1-1	1.1
Gfc①CP91076	T	C	T	G	G	1-1	5.6
Gfc①CP91077	G	C	C	G	G	1-1	2.5
Gfc①CP91078	T	C	C	G	G	1-1	1.4
Gfc①CP91079	G	C	C	G	G	1-2	4.3
Gfc①CP91082	T	C	T	A	T	1-1	ND
Gfc①CP91084	T	C	C	G	G	1-1	2.1
Gfc①CP91086	T	A	C	G	G	1-1	5.3
Gfc①CP91089	T	A	C	G	G	1-1	4.9
Gfc①CP91099	G	C	T	G	G	1-2	5.0

[a] : Markers were developed in previous study [1]. Single nucleotide polymorphisims were determined by Luminex. [b] : Mating type was determined by PCR with the primer fusALPHAfor and fusALPHArev for MAT1-1, and fusHMGfor and fusHMGrev for MAT1-2 [2]. [c] : Fumonisin was analyzed by RIDASCREEN®FAST Fumonisin Kit.

Table S2. Primer used for RT-PCR

Gene	Primer Sequence (5'- -3')				Size of expected amplification (bp)
	Forward		Reverse		
FUM21	HS919	GCAATCAGTGCCAAAAATCAT	HS920	TGTGGATCCGAACCATCAAT	282
FUM1	HS921	TGCAGAGAAGTACATCTCGAA	HS922	ACGTTGTCGATAATGGCGTT	290
FUM6	HS923	TGTCTTTGGAAACGGGCAT	HS924	TCTCCATTCTTCATCACGCT	225
FUM8	HS925	GACGATGCCGAGCAATGAT	HS926	TTTGGAACAGCTCCGGACAA	241
FUM10	HS927	ACGATGGCAAGGCCAATAAT	HS928	AGCATTTCTCCCAAATGGGT	228
Histone H3	HS899	TCTACCGGAGGTGTCAAGAA	HS801	AATCTCACGGACCAGACGCT	132

Table S3. Plasmid created in this study

Region in the FUM cluster (size)	Including gene	Primer Sequence (5'- -3') used for PCR amplification				Plasmid			
		Forward		Reverse		Before insertion of PCR product[a]	After insertion of PCR product[a]	Antibiotic resistance gene[b]	
								for *Escherichia coli*	for *Fusarium fujikuroi*
T21 (6.0kb)	*FUM21*	HS685	ATGCGGCCGCTCCTCCACCAGATGATGACA	HS686	ATGCGGCCGCGGGTGATTCGATTACTACCA	pCB1004	pCBT21KOD-2	CmlR	HygR
same as above	same as above	same as above		same as above		pDNAT1	pDT21-1	AmpR and KanR	NouR
	FUM21 with G888C substiution	HS748[c]	CATGGGGCACAGAGAATTTCATCATACAAAGTTTATTTCTCGATG	HS749[c]	CATCGAGAAATAAACTTTGTATGATGAAATTCTCTGTGCCCCATG	pDT21-1[d]	pDT21G888C-1[e]	AmpR and KanR	NouR
	FUM21 with G2551T substition	HS746[c]	CTGTAGATGAGGTCACAGAATTATGAAAATTGGGAAGACAGATAATG	HS747[c]	CATTATCTGTCTTCCCAATTTTCATAATTCTGTGACCTCATCTACAG	pDT21-1[d]	pDT21G2551T-2[e]	AmpR and KanR	NouR
T1 (9.3kb)	*FUM1*	HS489	ATGCGGCCGCAGATATAGACGTTCCTGTAG	HS532	ATGCGGCCGCTGTATGATGCAGGAGCATTT	pCB1004	pCBT1KOD-1	CmlR	HygR
T67 (6.1kb)	*FUM6, FUM7*	HS491	ATGCGGCCGCAGAMTGATGGTGATGCGTA	HS687	ATGCGGCCGCAAGGTATTAGAGATTCTGTC	pCB1004	pCBT67KOD-1	CmlR	HygR
	FUM6, FUM7					pBSNII99-3	pBSNT67-1[f]	AmpR	GenR
	FUM6 (*FUM7* in pBSNT67-1 was dysfunctioned by a nucleotide inser	HS949[c]	CGGCAAACTCCCGGTAACCCTAAGCCGTG	HS950[c]	CACGGCTTAGGGTTACCGGGAGTTTGCCG	pBSNT67-1[d]	pBSNT67046T-3[f]	AmpR	GenR
	FUM7 (*FUM6* in pBSNT67-1 was dysfunctioned by a nucleotide inser	HS947[c]	ATCGAAAATGTAGCCTTGCCATTAGCTGGGGAATCT	HS948[c]	AGATTCCCCAGCTAATGGCAAGGCTACATTTTCGAT	pBSNT67-1[d]	pBSNT67141A-3[f]	AmpR	GenR
T8310 (7.3kb)	*FUM8, FUM3, FUM10*	HS688	ATGCGGCCGCTTCTGTACATGGCAGCATAG	HS689	ATGCGGCCGCTGATGGAGGGTTTGGACGTA	pCB1004	pCBT8310KOD-1	CmlR	HygR
T11213 (5.9kb)	*FUM11, FUM12, FUM13*	HS690	ATGCGGCCGCCCTAAGGTCTTGTTTGTTCA	HS691	ATGCGGCCGCCTTGTTGGACTCGGATAGCT	pCB1004	pCBT11213KOD-1	CmlR	HygR
T141516 (7.5kb)	*FUM14, FUM15, FUM16*	HS692	ATGCGGCCGCACGACAGTGGAAGTCAGGTT	HS693	ATGCGGCCGCGATTGTCAATGTCCACAAGC	pCB1004	pCBT141516KOD-1	CmlR	HygR
T831011213 (12.8kb)	*FUM8, FUM3, FUM10, FUM11, FUM2, FUM13*	HS706	ATGCGGCCGCGGAATTCAATGTGTCAATAG	HS707	ATGCGGCCGCAGAAGTTTACGCTTCTTGTT	pBSNII99-3	pBSNT831011213KOD-5	AmpR	GenR

[a] : pCB1004 [3], pDNAT1 [4] and pBSNII99-3 were used. pBSNII99-3 was created by insertion of geneticin resistance cassette that cut out with *Eco* RV from pBS99 into *Nae* I site of pBluescript II KS(+)(Agilent Technologies). pBS99 was created by insertion of geneticin resistance cassette from pII99 [5]. Geneticin resistance cassette was cut out with *Bgl* II and *Xba* I from pII99 though *Xba* I terminal was blunted before *Bgl* II digestion. pBS99 was created by insertion of this geneticin resistance cassette at *Bam* HI and *Sma* I site of pBluescript II KS(+). [b] : Antibiotic resistance gene; Chloramphenicol resistance (CmlR), Ampicillin resistance (AmpR), Kanamycin resistance (KanR), Hygromycin resistance (HygR), Geneticin resistance (GenR), Nourseothricin resistance (NouR). [c] : Primer was used for creation of point mutation by QuickChange II XL Site-Directed Mutagenesis Kit (Stratagene). [d] : Plasmid was used for creation of point mutation by QuickChange II XL Site-Directed Mutagenesis Kit (Stratagene). [e] : Point mutation in the plasmid was created by QuickChange II XL Site-Directed Mutagenesis Kit (Stratagene). [f] : T67 fragment in pCBT67KOD-1 was cut out with *Not* I digestion and pBSNT67-1 was created by insertion of the fragment to *Not* I site in pBSNII99-3.

Table S4. SNP at 2551st in *FUM21*

Strain	Fumonisin producibility	g.2551G>T	Reference
Gfc0801001	Non-producer	T	This study
GL-24	Non-producer	T	This study
Gfc0625008	Non-producer	T	This study
Gfc1034001	Non-producer	T	This study
Gfc0825009	Producer	G	This study
Gfc0821004	Producer	G	This study
Gfc0009063	Producer	G	This study
41-79	Producer	G	This study
IMI58289	Low-producer	G	[6]
m567	Non or Low-producer	G	[6]
MRC227	Non or Low-producer	G	[6]
E282	Non or Low-producer	G	[6]
C1995	Non or Low-producer	T	[6]
B20	Non or Low-producer	T	[6]
FSU48	Non or Low-producer	T	[6]
NCIM 1100	Non or Low-producer	T	[6]
B14	Producer	G	[6]

Table S5. Primer used for Luminex assay

Primer sequence (5'- -3')				Objective	Reference
Forward		Reverse			
HS438	GTGTCAAACTAAACATTCGACAATAGGAAG	HS439	ATGATATGTTAGTATGAATAAGTAGAATTA	PCR for Luminex ASPE of TEF_T618G	[6]
HS641	TACACTTTCTTTCTTTCTTTCTTTGTATGAATAAGTAGAATTAC			Luminex ASPE of TEF_T618G, signal detection with LUA-12 (FlexMAPxtag)	[1]
HS642	CTTTTCATCAATAATCTTACCTTTGTATGAATAAGTAGAATTAA			Luminex ASPE of TEF_T618G, LUA-65	[1]
HS398	TTGGAAGTGGCCTACGAGTGT	HS399	GAAGATGGCATTGATTGCCT	PCR for Luminex ASPE of FUM1_G423A	[1]
HS540	TCATTTACTCAACAATTACAAATCCGGGCCAAACCGATTGGCGG			Luminex ASPE of FUM1_G423A, LUA-67	
HS541	AATCTAACAAACTCATCTAAATACCGGGCCAAACCGATTGGCGA			Luminex ASPE of FUM1_G423A, LUA-76	
HS506	ATGCGGCCGCGGRCCGAAAGAAGCATCCGA	HS519	AGCCAAAGAAACTTGGCAGT	PCR for Luminex ASPE of FUM18_G51T	[1]
HS542	AAACTAACATCAATACTTACATCACCGGAATCTTACGGCCTTTG			Luminex ASPE of FUM18_G51T, LUA-87	
HS543	AATCTCATAATCTACATACACTATCCGGAACCTTACGGCCTTTT			Luminex ASPE of FUM18_G51T, LUA-97	
P138-5	AACCCCTACATTGCCCCTATC	HS556	GGTTCATCTCAGCCTTGATA	PCR for Luminex ASPE of CPR_C1152A	[1], [7]
HS557	AATCAATCTTCATTCAAATCATCATCATCGTTGGGAGCAAATGCG			Luminex ASPE of CPR_C1152A, LUA-16	[1]
HS558	CTACAAACAAACAAACATTATCAATCATCGTTGGGAGCAAATGCT			Luminex ASPE of CPR_C1152A, LUA-28	[1]
P450-4-GD1	TTTCTCGGTCCAGAGCACTGCCGC	P450-4-GD2	CGTGGTCTTCCTTTCCCATCTGGC	PCR for Luminex ASPE of P4504_C842T	[8]
HS559	CTTTATCAATACATACTACAATCATCCGCGTTGTCCCCCATATC			Luminex ASPE of P4504_C842T, LUA-2	[1]
HS560	CTACTATACATCTTACTATACTTTTCCGCGTTGTCCCCCATATT			Luminex ASPE of P4504_C842T, LUA-14	[1]

Table S6. Primer used for sequencing of FUM cluster

Strain	Amplification fragment (size)	Primer [a]	Sequence (5'- -3')	Reference
Gfc0825009	A (8.8kb)	HS399*	GAAGATGGCATTGATTGCCT	[1]
		HS487*	ATGCGGCCGCTCTTGKGAYKWTTYGYMWT	
				A-fragment was obtained by PCR with HS399 and HS487 primer but the PCR product had HS399 sequence at both terminal. Then, A-fragment was cloned into pCR4-TOPO (Invitrogen) and terminal regions were sequenced with M13M4 (5'-GTTTTCCCAGTCACGACGTTGTAAA-3') and M13RV (5'-CAGGAAACAGCTATGACCATGATTA-3') primer.
		HS489	ATGCGGCCGCAGATATAGACGTTCCTGTAG	
		HS561	TGGTGCTTCGGTTGGTGAAT	
		HS562	CAGGTCAAAATACGGCTTCG	
		HS563	CGGAATCGTATAAATCTTTG	
		HS574	GCGACAAAACCATCAAAAGT	
		HS585	ATCGTTCTTT ACTCGATCCG	
		HS586	GCTATCACAA TGCTTCAGA	
		HS599	TTGCACTGTGCAGTTCCAAT	
		HS600	GAATCGGGATTGTTGAGAAG	
		HS607	ACTATCAAGACATCGAGAAA	
		HS608	TTGCTGAAGATCGTGGTCCT	
		HS617	ATCTGACTTGGCACTGATTG	
		HS618	GTAGGCTCTGACTACAGACA	
		HS621	TAGGCAATATAACAGCAGAT	
		HS622	GACACGTAATCGCTTCTCTC	
	B (7.9kb)	HS398*	TTGGAAGTGGCCTACGAGTGT	[1]
		HS532*	ATGCGGCCGCTGTATGATGCAGGAGCATTT	
		HS589	CATGGAACCGGTACATCCGT	
		HS590	GCCAGAGTTGAGAATCTCCA	
		HS623	TCCAGTTGATGCAATTCCCT	
		HS624	ATCTTTAGGTGCAGCGAGAT	
		HS635	TTATCCAGGTCTGCGGAATC	
		HS636	CTAGCCAGGTCAATACCAAT	
		HS643	TCGCTGGTGCTATGAAGCTT	
		HS644	TGAAGCCCGCTAACACTTGC	
		HS648	ACACAATGGCACCAGCTGGA	
		HS649	GCGTCACGAGTTCAAGCAGA	
		HS652	GGTAGAGCGACTTACGCTAC	
		HS653	TGCCTTGAATCGTTCCTGAG	
	C (4.6kb)	HS491*	ATGCGGCCGCAGAMTGATGGTGATGCGTA	
		HS494*	ATGCGGCCGCGTGGAACTGGTGATGATCAA	
		HS591	GCCATACAACCTGCTATTAC	
		HS592	AAGCGTATACTCTGCCAATG	
		HS609	CTGTAGAAAGGCTCTAAACG	
		HS610	GTCTACGATGGGCACGATTC	
		HS625	CCGATAGGTGCATCCAATGG	
		HS626	GACATTGCGGCTACGTGATC	
	D (3.1kb)	HS533*	ATGCGGCCGCAGCTGAAAGACTGGTCTCAA	
		rp680*	GCAAGCTTTGTGGCTGATTGTC	[9]
		HS564	CCGATTTCGTCGTCTTGGAC	
		HS565	AGCAAGCGTCAAGGCTTTCT	
		HS576	TTCCATCAGATAGCTGATAA	[1]
		HS577	GACAGCAGGGGTCTTGGAAA	[1]
	E (5.9kb)	rp679*	CGTAGTAGGATGAGAAGGATG	[9]
		HS534*	ATGCGGCCGCGTGTGTCATGTGAGTTGAAG	
		HS566	GATTCAGTGTTCCTAGAGCA	
		HS567	TGACCAAGCTGTCTCTAGAT	
		HS580	CTGGAGCTTGCAGCTCACT	
		HS581	TTGGGTTCGGAGCTCGATGC	
		HS582	GCATCTGGCTGCTTTGCTCT	
		HS583	ACTTTGTCCTCTGCCATAAC	
		HS584	ATCGTCAGCTCTTGCGTTGA	
		HS587	GACTTTGTTGACAATTTCAC	
		HS588	AACGATACTTCCGAGCACTG	
		HS603	TATGACCGTTATAGGCATAG	
		HS604	ATGCCAGTTTCGCAGTGTCT	
	F (4.8kb)	HS497*	ATGCGGCCGCGTCAACGATGCTGCTTGCTT	
		HS500*	ATGCGGCCGCGCACAGCATAGCCCACATGT	
		HS548	CTTTCTCATATACCATAAACA	
		HS549	TTGATCCAGGAGCCAGTTTC	
		HS552	GCATACGAATCCTGAATCGT	
		HS553	TACCAGCGATCATGAAGATG	
		HS554	AGAATACCGGACCGAGATGC	
		HS555	TCTGGCGCGATACGCACGAT	
		HS575	AAATGGTGACGCATATGCTT	
	G (7.5kb)	HS546*	ATGCGGCCGCACCTTCTCAGTTTTCTATGT	
		HS547*	ATGCGGCCGCTCGATTGCTCTGGTATCGTT	
		HS593	GTGGGCTATGCTGTGCTTGT	
		HS594	CGATTGTCATCATTATGCCA	
		HS611	GGAAAGGCATCAGACTCGAG	
		HS612	AATTGAGAACCAGAGTAGCT	
		HS627	ATGCTGATATATCTTTCTCA	
		HS628	GGCTGTTGTCTCATGCCCAG	
		HS637	ATGTCCTTGAGACCAGAGTG	
		HS638	TGGAATTTATGCTCGCTCCC	
		HS645	ACCGACATTGCCATTTAAGC	
		HS646	AAGAGAAACAGCAGTAGTGT	
	H (1.7kb)	HS544*	ATGCGGCCGCTTCAGTCGCTTCAGGGTTCT	
		HS536*	ATGCGGCCGCATTCCATTTACCTTGGTTAC	
		HS595	TCAGCTCTCCTTCGCTATAA	
		HS596	TTGATCCGCATCTCCTGGAC	

Strain	Amplification fragment (size)	Primer [a]	Sequence (5'- -3')	Reference
	H (1.7kb)	HS544*	ATGCGGCCGCTTCAGTCGCTTCAGGGTTCT	
		HS536*	ATGCGGCCGCATTCCATTTACCTTGGTTAC	
		HS595	TCAGCTCTCCTTCGCTATAA	
		HS596	TTGATCCGCATCTCCTGGAC	
	I (3.7kb)	HS503*	ATGCGGCCGCATGACGACGAGGAAGACTCT	
		HS506*	ATGCGGCCGCGGRCCGAAAGAAGCATCCGA	[1]
		HS597	GTCCAATCCAACGTTTTCAG	
		HS598	GACTTCGGATGCATCGCACT	
		HS613	GACTTCCAAATCACTCAACT	
		HS614	TGGATACAGCAAGTCATCTC	
	J (4.9kb)	HS511*	ATGCGGCCGCATTAWTTAGACYCTTGACGC	
		HS537*	ATGCGGCCGCTGCGATAAGCTTATTGCTAT	
		HS507	ATGCGGCCGCGCGTATCAAGTTCAAGAAAT	
		HS568	CATCCTCATCCCTCGAGACT	
		HS569	GAAGGTCTATACTGCGATTG	
		HS570	GAACTACGCAAAGCAGTCTT	
		HS571	CAAGATCGAAGGGTCAACAT	
		HS578	AGGTGCGCTACCTGGAGGTT	
		HS579	ACAGCTCATTCTGTACCAGA	
	Franking region (1.0kb)	HS470	GTAATACGACTCACTATAGGGCACGCGTGGTCGACGGCCCGGGCTGGT	Adapter for dual-suppression-PCR [10]
		HS471	ACCAGCCC-NH_2	Adapter for dual-suppression-PCR [10]
		HS472	CCATCGTAATACGACTCACTATAGGGC	AP1 for dual-suppression-PCR [10]
		HS473	CTATAGGGCACGCGTGGT	AP2 for dual-suppression-PCR [10] and sequencing
		HS572	TTCTTCTACTCGATGAGCCT	IP1 for dual-suppression-PCR [10]
		HS573	GACAGAAGGGATAATGCACA	IP2 for dual-suppression-PCR [10] and sequencing
Gfc0801001	K (8.0kb)	HS574*, HS563*, HS586, HS600, HS608, HS618, HS622, HS617, HS607, HS599, HS585, HS561, HS489, HS562	see above	
	L (8.5kb)	HS562*, HS532*, HS398, HS589, HS623, HS635, HS643, HS648, HS652, HS649, HS644, HS636, HS624, HS590	see above	
	M (6.5kb)	HS491*, HS577*, HS591, HS609, HS625, HS626, HS610, HS592, HS533, HS494, HS564, HS576	see above	
	N (5.5kb)	HS564*, HS587*, HS576, HS565, HS566, HS582, HS584, HS588, HS604	see above	
		HS660	CAAGCACTAAGTAGTGAGAG	
	O (7.0kb)	HS500*, HS583, HS581, HS575, HS548, HS552, HS554, HS555, HS553, HS549	see above	
		HS662*	ACTGCTCAGCCAGTAGGCAT	
		HS663	TGGCCTTGATTGGAAGAGTC	
		HS666	GTATTACTCCGAATTGAACT	
	P (7.5kb)	HS546*, HS547*, HS593, HS611, HS627, HS637, HS645, HS646, HS638, HS628, HS612, HS594	see above	
	Q (5.0kb)	HS544*, HS506*, HS503, HS536, HS597, HS613, HS614, HS598	see above	
		HS670	CCAGTACGAACAGCTCACCA	
	R (5.0kb)	HS537*, HS568, HS569, HS507, HS578, HS579, HS571, HS511	see above	
		HS677*	GTTGCAGCTAGGCAAGGTCT	

[a] : * indicates primers were used for PCR amplification and sequence.

Table S7. Transformants created in this study

Original strain (strains used for transfromation with the plasmid)	Plasmid used for transformation	Created transformant (after transfromation with the plasmid)	*FUM* gene(s) of Gfc0825009 integrated in Gfc0801001	Antibiotic resistance of created transformant[a]	Fumonisin positive transformant/investigated transformant[b]
Gfc0801001	pCBT21KOD-2	FfT21FUMKOD	*FUM21*	HygR	0/10
Gfc0801001	pCBT1KOD-1	FfT1FUMKOD	*FUM1*	HygR	0/5
Gfc0801001	pCBT67KOD-1	FfT67FUMKOD	*FUM6*, *FUM7*	HygR	0/3
Gfc0801001	pCBT8310KOD-1	FfT8310FUMKOD	*FUM8*, *FUM3*, *FUM10*	HygR	0/3
Gfc0801001	pCBT11213KOD-1	FfT11213FUMKOD	*FUM11*, *FUM12*, *FUM13*	HygR	0/3
Gfc0801001	pCBT141516KOD-1	FfT141516FUMKOD	*FUM14*, *FUM15*, *FUM16*	HygR	0/3
FfT67FUMKOD(#1)	pBSNT831011213KOD-5	FfT67831011213	*FUM6*, *FUM7*, *FUM8*, *FUM3*, *FUM10*, *FUM11*, *FUM2*, *FUM13*	HygR, GenR	0/14
FfT67831011213(#30)	pDT21-1	FfDTFUM21_6_13	*FUM21*, *FUM6*, *FUM7*, *FUM8*, *FUM3*, *FUM10*, *FUM11*, *FUM2*, *FUM13*	HygR, GenR, NouR	10/10
FfT67831011213(#30)	pDT21G888C-1	FfDTFUM21G888C_6_13	*FUM21* with G888C substitution, *FUM6*, *FUM7*, *FUM8*, *FUM3*, *FUM10*, *FUM11*, *FUM2*, *FUM13*	HygR, GenR, NouR	5/18
FfT67831011213(#30)	pDT21G2551T-2	FfDTFUM21G2551T_6_13	*FUM21* with G2551T substitution, *FUM6*, *FUM7*, *FUM8*, *FUM3*, *FUM10*, *FUM11*, *FUM2*, *FUM13*	HygR, GenR, NouR	0/22
FfT67FUMKOD(#1)	pDT21-1	FfDT21T67FUMKOD	*FUM21*, *FUM6*, *FUM7*	HygR, NouR	19/20
FfT21FUMKOD(#2)	pBSNT67-1	FfT67T21FUMKOD2	*FUM21*, *FUM6*, *FUM7*	HygR, GenR	10/20
FfT21FUMKOD(#2)	pBSNT67046T-3	FfT6TT21FUMKOD2	*FUM21*, *FUM6*	HygR, GenR	0/20
FfT21FUMKOD(#2)	pBSNT67141A-3	FfT7AT21FUMKOD2	*FUM21*, *FUM7*	HygR, GenR	18/20

[a] : Antibiotic resistance gene; Chloramphenicol resistance (CmlR), Ampicillin resistance (AmpR), Kanamycin resistance (KanR), Hygromycin resistance (HygR), Geneticin resistance (GenR), Nourseothricin resistance (NouR).

[b] : Fumonisin was analysed by RIDA SCREEN FAST Fumonisin Kit.

References

1. Suga, H.; Arai, M.; Fukasawa, E.; Motohashi, K.; Nakagawa, H.; Tateishi, H.; Fuji, S.; Shimizu, M.; Kageyama, K.; Hyakumachi, M. Genetic differentiation associated with fumonisin and gibberellin production in Japanese *Fusarium fujikuroi*. *Appl. Environ. Microbiol.* **2019**, *85*, doi:10.1128/AEM.02414-18.
2. Kerenyi, Z.; Moretti, A.; Walwijk, C.; Olah, B.; Hornok, L. Mating type sequences in asexually reproducing *Fusarium* species. *Appl. Environ. Microbiol.* **2004**, *70*, 4419–4423.
3. Carroll, A.M.; Sweigard, J.A.; Valent, B. Improved vectors for selecting resistance to hygromycin. *Fungal Genet. Newsl.* **1994**, *41*, 20–21.
4. Kück, U.; Hoff, B. Application of the nourseothricin acetyltransferase gene (*nat*1) as dominant marker for the transformation of filamentous fungi. *Fungal Genet. Rep.* **2006**, *53*, 9–11.
5. Namiki, F.; Matsunaga, M.; Okuda, M.; Inoue, I.; Nishi, K.; Fujita, Y.; Tsuge, T. Mutation of an arginine biosynthesis gene causes reduced pathogenicity in *Fusarium oxysporum* f. sp. *melonis*. *Mol. Plant Microbe Interact.* **2001**, *14*, 580–584.
6. Suga, H.; Kitajima, M.; Nagumo, R.; Tsukiboshi, T.; Uegaki, R.; Nakajima, T.; Kushiro, M.; Nakagawa, H.; Shimizu, M.; Kageyama, K. A single nucleotide polymorphism in the translation elongation factor 1α gene correlates with the ability to produce fumonisin in Japanese *Fusarium fujikuroi*. *Fungal Biol.* **2014**, *118*, 402–412.
7. Malonek, S.; Rojas, M.C.; Hedden, P.; Gaskin, P.; Hopkins, P. The NADPH-cytochrome p450 reductase gene from *Gibberella fujikuroi* is essential for gibberellin biosynthesis. *J. Biol. Chem*. **2004**, *279*, 25075–25084.
8. Malonek, S.; Rojas, M.C.; Hedden, P.; Gaskin, P.; Hopkins, P.; Tudzynski, B. Functional characterization of two cytochrome P450 monooxygenase genes, *P450-1* and *P450-4*, of the gibberellic acid gene cluster in *Fusarium proliferatum* (*Gibberella fujikuroi* MP-D). *Appl. Environ. Microbiol.* **2005**, *3*, 1462-1472.
9. Proctor, R.H.; Plattner, R.D.; Brown, D.W.; Seo, J.-A.; Lee, Y-W. Discontinuous didtribution of fumonisin biosynthetic genes in the *Gibberella fujikuroi* species complex. *Mycol. Res.* **2004**, *108*, 815–822.
10. Lian, C.; Hogetsu, T. A protocol for efficient development of microsatellite markers. *J. Jpn. For. Soc.* **2004**, *86*, 191–198. (In Japanese)
